# Metronidazole Induced Cerebellar Toxicity: A Case Report

**DOI:** 10.31729/jnma.6644

**Published:** 2021-09-30

**Authors:** Shova Sapkota, Aliska Niroula, Rina Prajapati, Subhani Sharma, Krishna Dhungana

**Affiliations:** 1 Kathmandu Medical College and Teaching Hospital, Sinamangal, Kathmandu, Nepal; 2 Department of Neurology, Kathmandu Medical College and Teaching Hospital, Sinamangal, Kathmandu, Nepal

**Keywords:** *case report*, *cerebellar toxicity*, *metronidazole*, *side effect*

## Abstract

Metronidazole is a widely used antibiotic against bacteria! and protozoan infections. Even though the therapeutic use of the drug is high, it is associated with some severe side effects like neurotoxicity such as optic neuropathy, peripheral neuropathy, encephalopathy and cerebellar toxicity. We present a case of a 55-years male presented with dysarthria, who had positive cerebellar sign and magnetic resonance imaging findings suggestive of metronidazole induced cerebellar toxicity following metronidazole therapy for two months in a case of liver abscess. And, the symptoms resolved after cessation of metronidazole.

## INTRODUCTION

Metronidazole is a nitroimidazole antibiotic, widely used against bacterial and protozoan infection. It is effective against amoebic liver abscess and is associated with side effects of mild to moderate gastrointestinal disturbances such as nausea, vomiting, abdominal pain and diarrhea but severe neurotoxicity such as optic neuropathy, peripheral neuropathy, encephalopathy and cerebellar toxicity have been reported in rare cases.^1^ Here, we present a case that presented with dysarthria, had positive cerebellar sign and magnetic resonance imaging findings suggestive of metronidazole induced cerebellar toxicity following metronidazole therapy for two months in a case of liver abscess. The symptoms resolved after cessation of metronidazole.

## CASE REPORT

A 55 years male presented to the neurology outpatient department of our hospital with the chief complaint of slurring of speech for 3 days which was acute on the onset and progressive in nature. He could understand the spoken or written language and could obey the command; there was also complaint of on and off dizziness. There was no history of headache, vomiting, blurring of vision, neck rigidity, facial deviation, difficulty in swallowing. He had no complaints of any abnormal body movements, focal weakness of any body parts, and stool or urinary incontinence. He was diagnosed with an amoebic liver abscess two months back for which he was taking tablet levofloxacin, metronidazole (400mg) thrice a day and combination of diloxanide furoate and metronidazole (500mg + 400mg) thrice a day for two months. He is a known case of type II diabetes mellitus for which he has been taking metformin 850mg twice a day since 3 years and hypertension for which he has been taking amlodipine 2.5mg for two months. He is an occasional smoker and social drinker.

**Figure 1:**
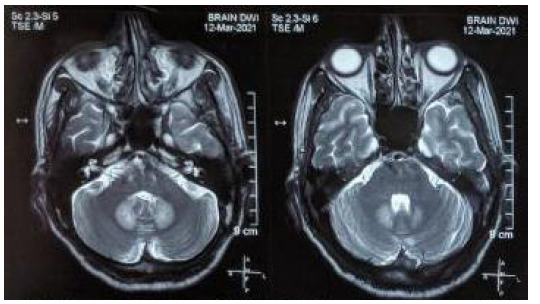
MRI of the brain showing symmetrical high signal intensity in the dentate nucleus of the bilateral cerebellum.

On examination, his blood pressure was 140/80 mmHg, pulse was 88 beats per minute, respiratory rate was 18 breaths per minute, SpO_2_ 96% on room air. His Glassgow Coma Score was 15/15, higher mental functions were intact, signs of meningeal irritations were absent and sensory and motor functions were intact. Cerebellar signs were elicited where dysdiadokinesia was present, tandem gait was impaired and Romberg's test was positive. Patient was admitted to the medicine ward with the impression of cerebellar stroke. He was given tablet aspirin 75mg per oral once a day, injection insulin 8 unit subcutaneous once a day, intravenous ondansetron 4mg, vitamin B12 1500mg per oral once a day and other supportive treatment while continuing the medicine he was taking before. On routine investigation his random blood sugar was 161mg/dl, electrolytes were within normal range and his blood cell counts were normal while hemoglobin was 11.8gm/dl. His liver function test was normal. For diagnosis of cerebellar stroke, computed tomography scan of head and bilateral carotid and vertebral artery doppler was done which showed normal findings. So, on the next day the Magnetic Resonance Imaging (MRI) of the brain was done. On plain MRI of the brain, there was symmetrical T2/FLAIR high signal intensity in the dentate nucleus of the bilateral cerebellum. With a supportive history of metronidazole intake, examination and MRI findings, the patient was diagnosed with metronidazole induced cerebellar toxicity (Figure 1).

After diagnosis, metronidazole, diloxanide furoate and levofloxacin were discontinued and tablet cefixime 200mg twice a day was given for treatment of liver abscess. Patient was also prescribed vitamin B12 1500mg, vitamin E 600mg supplementation once a day for one month at the time of discharge. After cessation of metronidazole intake, cerebellar signs resolved within two weeks. Follow up MRI was not done as the symptoms of cerebellar toxicity had completely resolved.

## DISCUSSION

Metronidazole is nitroimidazole antibiotic, used to treat anaerobic or microaerophilic microorganisms such as Entamoeba histolytica, Trichomonas vaginalis, Giardia lamblia, Clostridium difficile, Helicobacter pylori. Metronidazole is associated with mild to moderate side effects such as nausea, abdominal pain and diarrhea. More than 42g dose of metronidazole may increase the risk of peripheral neuropathy. Neurotoxicity such as cerebellar dysfunction, visual impairment, vestibulo-toxicity, cochlear toxicity, ataxic gait, dysarthria and seizures have been reported in some rare cases.^1^ High concentration of metronidazole in brain extracellular fluid contributes to central nervous system toxicity.^2^ In case with history of metronidazole intake and clinical suspicion, neuroimaging is the modality for diagnosis.^3^ Also, the signs and symptoms of toxicity resolves with cessation of metronidazole.^4^

A systematic review done by Kuriyama, et al. on metronidazole induced central nervous system toxicity, 77% of patients had cerebellar dysfunction, 33% had altered mental status and 15% had seizures. Average age of patients was 53.3 years and 64% of patients were male and median duration of metronidazole intake was 54 days. 65% had complete resolution of their symptoms with discontinuation of metronidazole therapy. Most patients presenting with cerebellar dysfunction had symptoms like dysarthria, ataxia, dysmetria and nystagmus in decreasing order.^5^ In our case, a 55 years male patient presented with cerebellar dysfunction such as dysarthria, dysdiadochokinesia, impaired tandem gait and positive Romberg's sign after taking metronidazole for two months and his symptoms resolved completely after cessation of treatment.

According to Kim, et al, in cases of metronidazole induced encephalopathy MRI demonstrates bilaterally symmetrical brain lesions at cerebellar dentate nuclei, dorsal medulla, dorsal pons, midbrain and splenium of corpus callosum.^6^ In our case, symmetrical hyperintense focus was noted in dentate nucleus of bilateral cerebellum. Similar finding was also reported by Agrawal, et al. in 80-years female with metronidazole induced cerebellar toxicity.^7^

Both clinical symptoms and MRI changes substantially resolve with cessation of metronidazole.^4^ In a case reported by Lefkowitz, et al, 59 years diabetic male with alcoholic liver cirrhosis showed complete resolution of both clinical and MRI features of metronidazole induced cerebellar toxicity after four weeks of cessation metronidazole therapy.^8^ In our case also patient was free of symptoms and signs of cerebellar toxicity after two weeks of discontinuation of metronidazole while MRI was not done during follow up.
